# Pituitary abscess: two case reports

**DOI:** 10.1186/s13256-019-2280-8

**Published:** 2019-11-24

**Authors:** Yaotse Elikplim Nordjoe, Suzanne Rita Aubin Igombe, Fatima Zahra Laamrani, Laila Jroundi

**Affiliations:** 1 Radiology Department, Centre Hospitalo-Universiataire Ibn Sina, Rabat, Morocco; 2 Temara, Morocco

**Keywords:** Pituitary abscess, MRI

## Abstract

**Background:**

Pituitary abscess is a rare condition with nonspecific symptoms that can be delayed. Proper diagnosis needs to occur preoperatively so that the management can be set up accordingly. Accurate diagnosis is challenging because many differential diagnoses can exhibit the same magnetic resonance imaging features.

**Case presentation:**

We report two cases of pituitary abscess. The first patient was a 66-year-old Arab woman who underwent a surgical procedure for a pituitary macroadenoma and presented 3 months later with chronic headaches and panhypopituitarism. A pituitary abscess was found on the follow-up magnetic resonance imaging. The second patient was a 64-year-old Arab man with no medical history who presented with a chiasmal syndrome with headaches and panhypopituitarism. Brain magnetic resonance imaging showed a heterogeneous pituitary mass that turned out to be a pituitary abscess intraoperatively. These two patients were treated with hormone substitution, endoscopic transsphenoidal drainage, and antibiotherapy, with excellent outcomes.

**Conclusions:**

Pituitary abscess is a rare and serious condition. Preoperative diagnosis can be challenging because of the many existing differential diagnoses upon imaging. Magnetic resonance imaging is the mainstay technique of imaging due to its multimodal nature. These cases demonstrate the variable patterns of a pituitary abscess seen on magnetic resonance imaging and the potential difficulties in achieving an accurate diagnosis preoperatively due to many other conditions potentially exhibiting the same magnetic resonance imaging features.

## Background

The pituitary abscess (PA) is a rare clinical entity, often with poor prognosis [1], accounting for less than 1% of all cases of pituitary lesions in specialized centers [2–4]. Preoperative accurate diagnosis of this condition is a crucial step in the planning of its management. Magnetic resonance imaging (MRI) is the best imaging technique available because of its multimodality [5]. However, the diagnosis of PA is often very challenging because many other pituitary conditions can present similar MRI features.

This report describes two cases of a rare condition and aims to serve an educational purpose for radiologists to increase their awareness of this condition, especially among patients who have risk factors.

## Case presentation

### Patient 1

A 66-year-old Arab woman with diabetes mellitus of 4 years’ duration underwent transsphenoidal (TSS) resection of a nonfunctioning pituitary macroadenoma in May 2018. Her immediate postoperative course was free of events. Three months after the procedure (August 2018), she checked in to our institution with chronic headaches. Her physical examination showed bitemporal hemianopia. Relevant blood screening results were as follows: thyroid stimulating hormone ultra sensible (TSHus), 0.3 mIU/L (normal range, 0.5–5); free thyroxine (FT4), 0.7 ng/dl (1–2); adrenocorticotropic hormone (ACTH) (8:00 a.m.), 3 pg/ml (10–40); cortisol (8:00 a.m.), 6 μg/dl (10–20); follicle stimulating hormone (FSH), 20 IU/L (30–110 postmenopausal); luteinizing hormone (LH), 10 IU/L (15–54 postmenopausal); estradiol, 1.2 pg/ml (< 10 postmenopausal); growth hormone (GH), 2 ng/ml (< 10 [women]); prolactin, 0.7 ng/ml (2–20 [nonpregnant]); C-reactive protein (CRP), 2 mg/L (< 3.0); and white blood cells (WBC), 10.5 × 10^9^/L (4.0–11.0 × 10^9^/L).

**Fig. 1:**
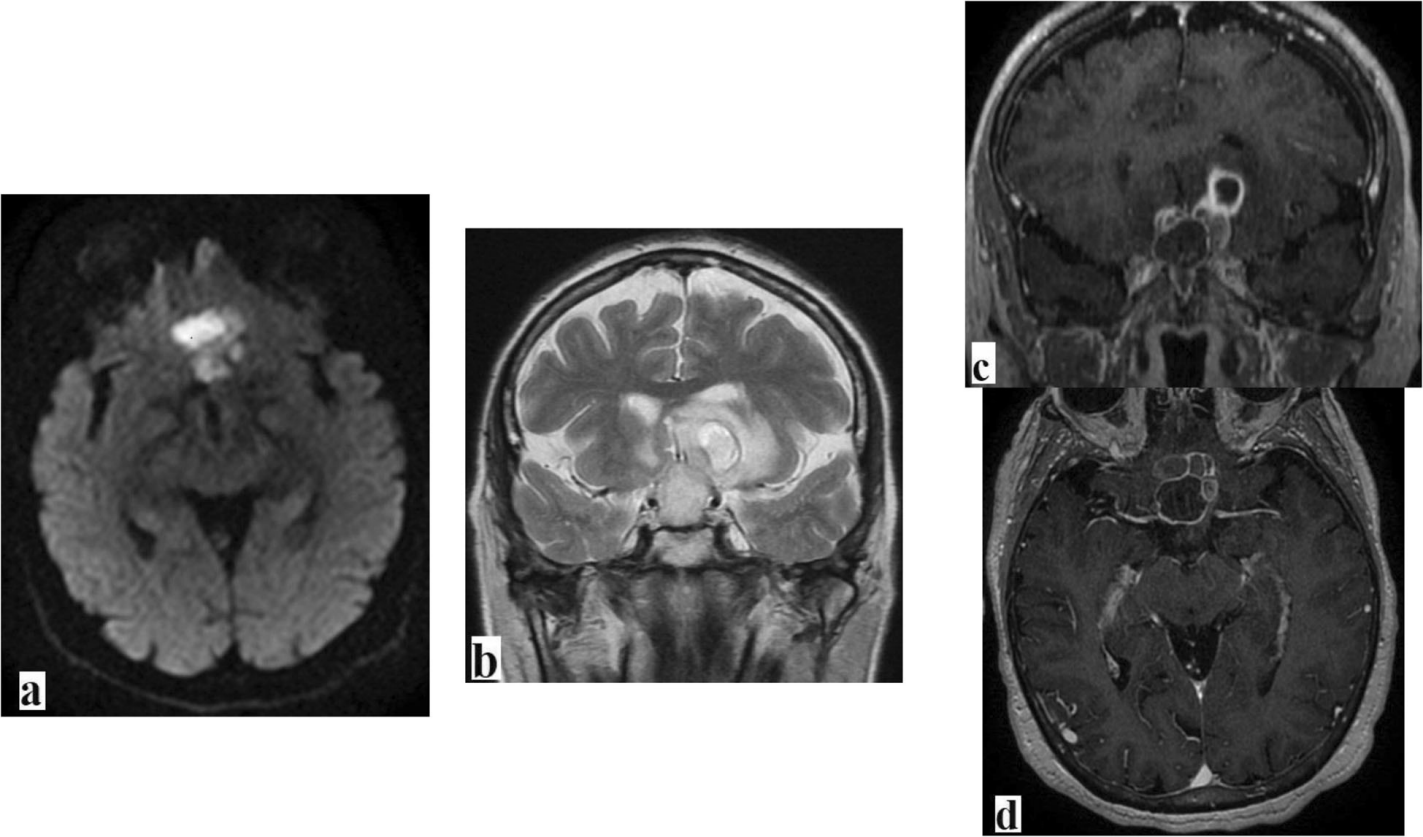
Brain magnetic resonance imaging scans. **a** Diffusion-weighted imaging (DWI). **b** T2-weighted (T2w) imaging. **c, d** T1-weighted (T1w) imaging + gadolinium. Images show a multilocular intra and suprasellar pituitary cystic mass exhibiting a typical rim enhancement pattern, with the central part being in high-intensity DWI/T2w and low-intensity T1w. This pituitary abscess is associated with peripheral edema (**b**) and is responsible for local compression mainly on the optical chiasma

Brain MRI showed a pituitary mass measuring 4 cm of the major axis, exhibiting low T1-weighted (T1w) signal, high T2-weighted (T2w) signal, and diffusion-weighted imaging (DWI) with rim enhancement after injection of gadolinium. The mass was responsible for a compression of the optical chiasma (Fig. 1).

**Fig. 2:**
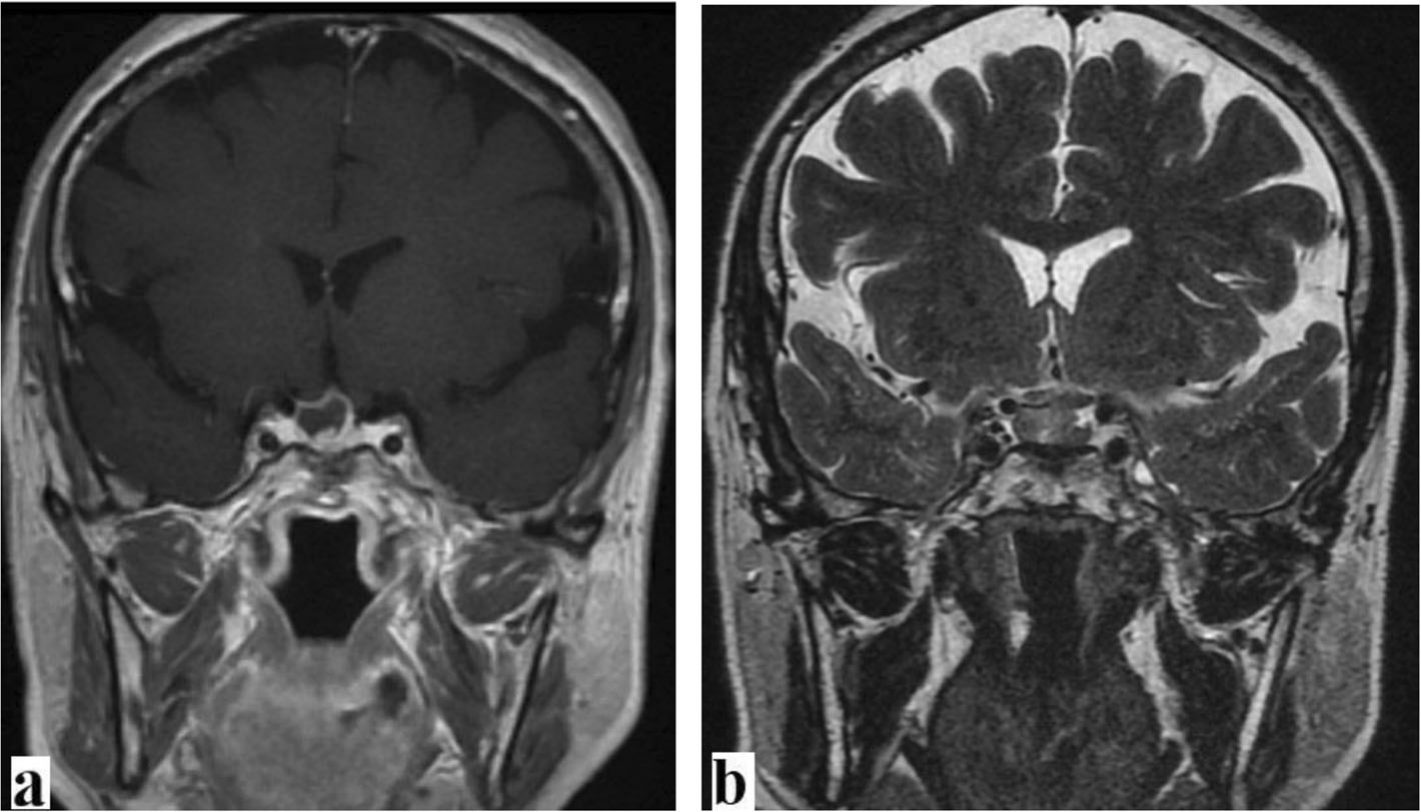
Same patient as in Fig. 1. Follow-up magnetic resonance imaging 3 months after the treatment showed a very sizable reduction of the pituitary abscess with disappearance of the peripheral edema and the local compression effect. **a** T1-weighted image + gadolinium. **b** T2-weighted image

After hormone substitution therapy, endoscopic endonasal drainage via TSS route was performed, which brought back a purulent fluid with no macroscopic tissue fragments inside. Following the drainage and irrigation of the PA, the result of a close exploration was negative for tumoral lesion. No intraoperative cerebrospinal fluid (CSF) leak in conjunction with a valsalva maneuver. Closure was achieved with Gelfoam packing (Pharmacia and Upjohn, Kalamazoo, MI, USA) and Surgicel overlay (Ethicon, Somerville, NJ, USA). The purulent material grew a *Streptococcus epidermidis*, and histopathological examination showed no tumoral lesion. On the basis of the antibiotic sensitivity test, targeted antibiotherapy was administered for 3 weeks. The postoperative course was uneventful, with progressive improvement of the clinical state. Follow-up MRI at 3 months showed a very sizable reduction of the PA (Fig. 2).

### Patient 2

A 64-year-old Arab man with no major medical history consulted in December 2017 for chronic headaches and progressive vision loss, evolving for 1 year, with neither fever nor asthenia. His physical examination was remarkable only for a chiasmal syndrome. He did not have diabetes insipidus. Relevant blood screening results were as follows: TSHus, 0.25 (0.5–5); FT4, 0.7 ng/dl (1–2); ACTH (8:00 a.m.), 5 pg/ml (10–40); cortisol, 3 μg/dl (10–20); FSH, 0.7 IU/L (1–10); LH, 0.5 IU/L (0.7–7.9 [ages 20–70 years]); testosterone, 50 ng/dl (200–900 [male age > 19]); GH, 1.5 ng/ml (< 5 [men]); CRP, 3 mg/L (< 3.0); and WBC, 9.7 × 10^9^/L (4.0–11.0 × 10^9^/L).

**Fig. 3:**
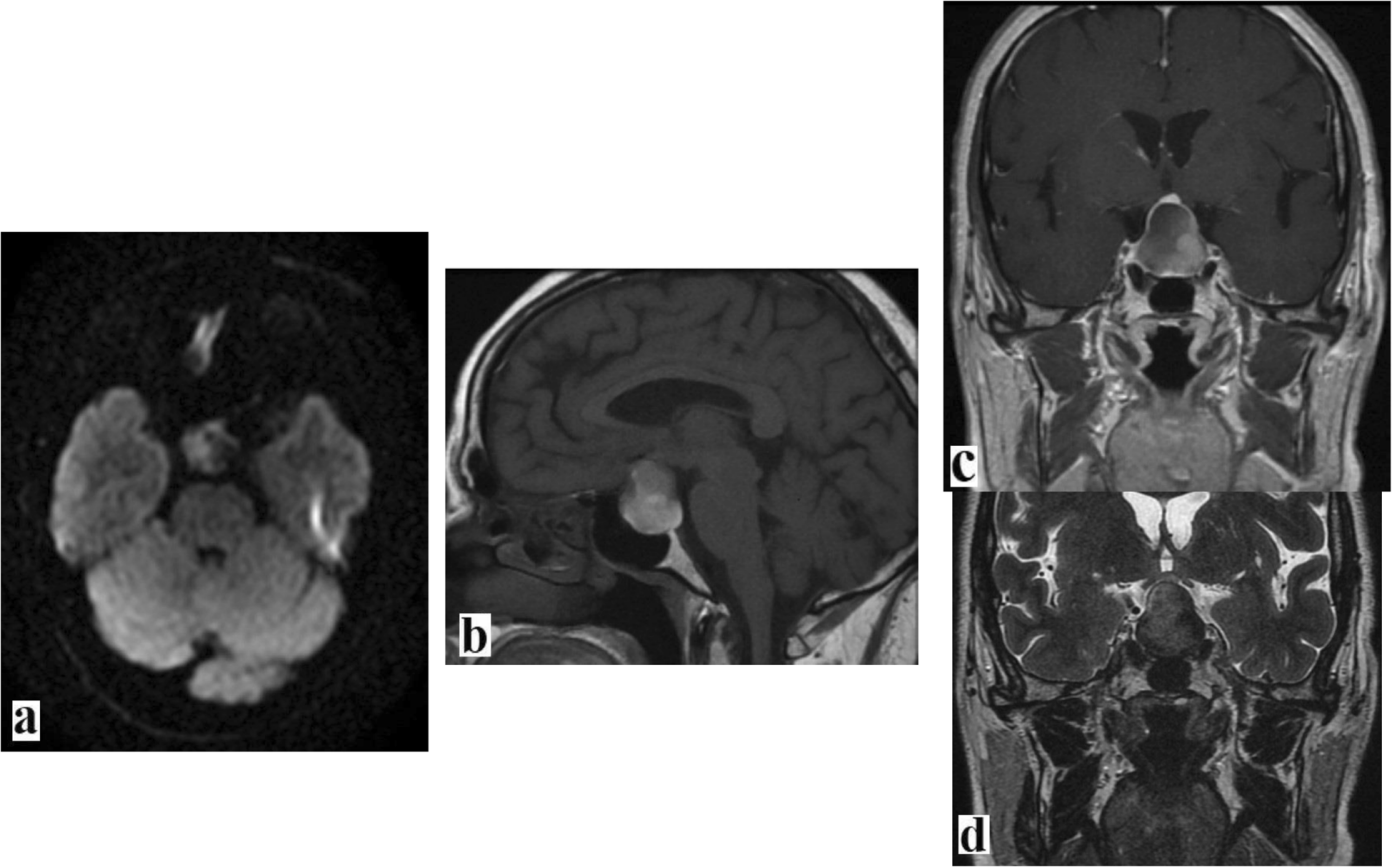
Brain magnetic resonance imaging scans. **a** Diffusion-weighted imaging (DWI). **b** T1w-weighted (T1w) imaging. **c** T1w + gadolinium. **d** T2-weighted (T2w) imaging. Images show an enormous pituitary mass exhibiting atypical features such as a heterogeneous mixed high- and low-signal T1w and T2w with rim enhancement. DWI showed a mild and partial high intensity in the central part of the mass. These features suggest a mixed cystic and hemorrhagic nature of this pituitary abscess

Brain MRI showed a 3-cm pituitary mass with a cystic and hemorrhagic component; it was heterogeneous with mixed high and low T1w and T2w signaling, and it exhibited rim enhancement. DWI showed mild and partial high intensity in the central part of the mass (Fig. 3).

At this stage, the two relevant differential diagnoses were pituitary adenoma (with necrosis and cyst degeneration) and PA. These MRI findings were mostly in favor of a remodeled pituitary mass. That is what was shown on the final MRI report.

**Fig. 4:**
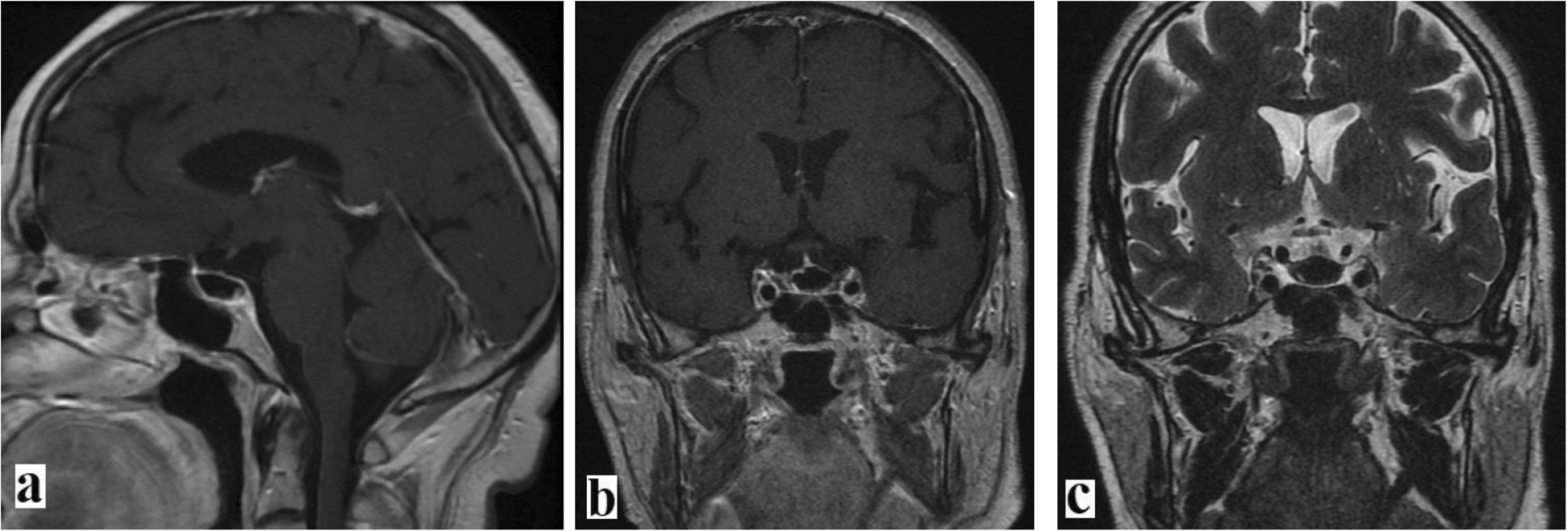
Same patient as in Fig. 3. Follow-up magnetic resonance imaging 3 months after the treatment showed complete drainage of the pituitary abscess, leaving as a sequela a cavity filled with air. **a, b** T1-weighted imaging + gadolinium. **c** T2-weighted imaging

After hormone substitution therapy, patient 2 underwent TSS surgery with intraoperative discovery of a PA that was drained. No macroscopic tissue fragments were found inside the purulent matter. Following drainage and irrigation of the PA, the result of a close exploration was negative for tumoral lesion. There was no intraoperative CSF leak in conjunction with a valsalva maneuver. Closure was achieved with Gelfoam packing and Surgicel overlay. The purulent material grew *Staphylococcus aureus*, and histopathological examination showed no tumoral lesion. On the basis of an antibiotic sensitivity test, targeted antibiotherapy was administered for 3 weeks. The postoperative course was uneventful, with progressive improvement of the clinical state. Follow-up MRI at 3 months showed complete drainage of the abscess (Fig. 4).

## Discussion and conclusions

PA is a rare condition, often with a poor prognosis. Only about 200 cases have been described in the literature to date [2, 6] since the first two cases described by Heslop in 1848 and Simmonds in 1914, respectively [7]. PA is classified as primary or *de novo* (developing in a previously normal pituitary gland), accounting for about 70% of cases, and secondary PA arising within preexisting pituitary lesions, accounting for 30% [8]. The primary subtype arises from hematogenous seeding of a systemic infection or local spread from a regional infection such as paranasal sinusitis (especially sphenoid sinusitis), meningitis, and cavernous venous thrombophlebitis [5, 8]. PA can develop secondary to preexisting conditions such as a pituitary adenoma, Rathke’s cleft cyst, craniopharyngioma, granulomatous disease, or autoimmune lymphocytic hypophysitis (in children) [9, 10, 11]. Secondary PA is also a well-known but rare complication of pituitary surgical procedures or radiation interventions [8, 12, 13]. PA is considered an infectious disease with a broad spectrum of causative microorganisms [2, 6, 8].

Many authors believe in the existence of an “abscess-sterile” entity or a “noninfectious PA” because the presentation does not always involve fever and high blood leukocyte count and because there is a high percentage of negative microorganism culture results. Although some authors believe that a sterile abscess is not a true abscess, but rather a liquefaction of necrosis of an infarcted pituitary gland or the contents of an atypical pituitary cyst, others maintain that a sterile culture may be due to an inadequate bacteriological technique in regard to fungus and anaerobic bacteria difficult to culture or to a broad use of antibiotic therapy initiated before or during surgery [8, 14].

The clinical presentation of PA is usually misleading because there are no specific symptoms. The symptoms are predominantly represented by headaches, pituitary hypofunction, visual disturbances, and an increased intracranial pressure syndrome, whereas infectious syndrome is often discreet and inconstant [2, 6, 8, 14]. Patients can present with acute, subacute, or deliberately chronic symptoms, thus explaining the late diagnosis in some cases.

An accurate diagnosis has to be ensured preoperatively so that management can be set up accordingly. Multimodal MRI is the mainstay imaging technique to ensure a proper diagnosis. However, despite all the advances in MRI, accurate diagnosis is challenging because many other differential diagnoses can exhibit the same MRI features, thus leading to the high rate of misdiagnosed PAs [5].

MRI scanning of PAs demonstrated an intrasellar/suprasellar (64.7%) or limited intrasellar (35.3%) cystic or partially cystic pituitary mass [11, 15–17]. The signal of the lesion depends on the composition of its content: the proportion of water, proteins, and lipids and whether hemorrhage is present. In a retrospective analysis of the MRI features of 51 PA cases by Wang *et al*. [5], PA neuroimaging revealed special radiological features on MRI, including low or isointense T1w (58.8%) signal, isointense or high T2w (76.5%) signal, and disappearance of the posterior pituitary bright spot in most cases. After gadolinium injection, rim or rimlike enhancement is noted in 82.4% of patients, half of whom showed typical rim enhancement (profound peripheral enhancement with internal hypointensity); others showed atypical rim enhancement with special signs, such as enhanced thick abscess wall and hyperintense flocculent or cotton-like foci within the internal hypointense region. Almost all patients presented at least one sign of adjacent anatomical structure invasion, including peripheral meningeal enhancement, pituitary stalk thickening, and paranasal sinus mucosal enhancement [5].

DWI is widely used to differentiate cerebral abscess from other necrotic masses [18]. Brain abscesses typically show high intensity on DWI with decreased apparent diffusion coefficient (ADC) value in their central region. However, the high intensity on DWI is not specific to PA, because pituitary apoplexy can also exhibit high intensity on DWI but normal to high ADC value due to the T2 shine-through effect [19]. The accuracy of DWI in PA remains controversial. In the Wang *et al*. case series, PA was misdiagnosed in one-third of the cases, which is one of the lowest rates of misdiagnosis of PAs in the literature [5].

In our patient 1, we did not have difficulties in achieving the diagnosis of PA, owing to the fact that classical MRI features were present. In addition, we were aided by the recent pituitary surgical procedure of the patient, because it represents a predisposing factor. However, in our patient 2, we could not achieve the proper preoperative diagnosis, because the patient had no relevant medical history, and his presentation on MRI was atypical. We associated these MRI characteristics with a pituitary adenoma with hemorrhagic necrosis and cystic degeneration. Accurate diagnosis was achieved intraoperatively. PAs can develop alongside or even complicating a local tumor, and close exploration after drainage and irrigation of the abscess showed no sign of tumoral mass in both of our patients.

The diagnosis of PA is challenging, partly because of its rareness (leading to the physician’s lack of experience) and because many other pituitary conditions can exhibit the same MRI features. All these factors contribute to the relatively high rate of misdiagnosis of PAs. Radiologists have to be aware of these intricacies and react accordingly by considering PA as a possible diagnosis, mainly in the presence of underlying conditions favorable to the development of a PA, such as a history of pituitary surgical or radiation interventions, growing local infection or mass, diabetes mellitus, and immunodeficiency. The main differential diagnoses are pituitary adenoma (with necrosis or cystic degeneration), pituitary apoplexy, Rathke’s cleft cyst, cystic craniopharyngioma, and lymphocytic hypophysitis (mostly in children) [5].

Regarding the treatment of PAs, although antibiotics alone could be successful [20], most authors consider prompt surgical exploration, either via a TSS or transcranial (TC) approach, to be the first choice [14, 21–24]. This is combined with culture of the purulent material and postoperative antibiotic therapy on the basis of antibiotic sensitivity test. Hormone replacement therapy is recommended whenever appropriate. TSS has the advantages of efficacy, safety, minimal invasiveness, and short hospital stay compared with TC surgery [22, 23]. Nevertheless, TC surgery is suitable when abscesses are suprasellar [22] or when the patient is not a candidate for TSS [21]. Our patients were treated with the combination of hormone substitution, TSS drainage, and antibiotic therapy, with an excellent outcome in both cases.

Prompt diagnosis and treatment of PAs yield a favorable prognosis. In the systematic review of the literature by Agyeis *et al*. [6], visual deficits improved in 75.7% of patients; 32.3% had complete recovery of endocrine function; 33.8% had partial recovery; and 22.5% had no recovery of pituitary function. The recurrence rate was about 10%, and the mortality rate was 4.5%.

In conclusion, PA is a very rare disease of the sellar region that is difficult to correctly diagnose on the basis of clinical manifestations and MRI. Despite advances in MRI, preoperative diagnosis of PA is still challenging to radiologists. MRI of PAs usually reveals a cystic or partially cystic pituitary mass, sometimes accompanied by some special signs. Despite its rareness, a PA must be considered in the case of a pituitary mass associated with intracranial hypertension, pituitary hormone dysregulation, and an infectious syndrome (which is a less reliable sign). In addition, there are some underlying predisposing factors, such as a history of pituitary surgical or radiation interventions, local infection or growing local mass, and diabetes mellitus. The treatment is a combination of surgery, antibiotics, and hormone substitution when needed. These cases demonstrate the variable patterns of PA in MRI and the potential difficulties in achieving an accurate diagnosis preoperatively, because many other pituitary conditions can exhibit the same MRI features. However, MRI remains the mainstay technique of imaging because of its multimodality. By associating these two cases, we aim to show a typical MRI finding of PA (patient 1) and an atypical one (patient 2). Radiologists have to be aware of the variable MRI patterns of this condition and deal with the findings accordingly.

## Data Availability

Not applicable.

## Abbreviations

ACTH: Adrenocorticotropic hormone
ADC: Apparent diffusion coefficient
CRP: C-reactive protein
CSF: Cerebrospinal fluid
DWI: Diffusion-weighted imaging
FSH: Follicle stimulating hormone
FT4: Free thyroxine
GH: Growth hormone
LH: Luteinizing hormone
MRI: Magnetic resonance imaging
PA: Pituitary abscess
T1w: T1-weighted
T2w: T2-weighted
TC: Transcranial approach
TSHus: Thyroid stimulating hormone ultra sensible
TSS: Transsphenoidal approach
WBC: White blood cells
